# An efficient method for tracking a magnetic target using scalar magnetometer array

**DOI:** 10.1186/s40064-016-2170-0

**Published:** 2016-04-22

**Authors:** Liming Fan, Chong Kang, Xiaojun Zhang, Quan Zheng, Ming Wang

**Affiliations:** College of Mechanical and Electrical Engineering, Harbin Engineering University, Harbin, 150001 China; College of Science, Harbin Engineering University, Harbin, 150001 China

**Keywords:** Tracking, Real-time, Scalar magnetometer array, Geomagnetic field intensity, Magnetic anomaly

## Abstract

The position of a magnetic target can be obtained through magnetic anomaly which is measured by a magnetic sensor. Comparing with vector magnetic sensor, the measurement value of the scalar magnetic sensor is almost not influenced by its orientation in measurement coordinate axes. Therefore, scalar magnetic sensors can be easily assembled into an array. Based on analysis of the total scalar magnetic anomaly measured by scalar magnetometer, we present an efficient method for tracking a magnetic target using scalar magnetometer array. In this method, we separate the position information and magnetic moment information of magnetic target by matrix transformation. Then, we can obtain the position of the magnetic target in real time by a scalar magnetometer array and a particle swarm optimization algorithm. In addition, the magnetic moment of the target can be estimated when the target’s position had been calculated. The simulation shows that the position of the target can be calculated accurately and the relative error of the position is <5 %. The calculated magnetic moment of the target is close to the theoretical value. In addition, execution time of each calculation is <1 s. Thus, the position of the magnetic target can be obtained in real-time through this method.

## Background

It is well known that magnetic object like unexploded ordnance (UXO) or underwater vehicle can be detected by magnetic sensors. There are different ways for localization or track of the magnetic object through magnetic anomaly (Nara et al. 2006; McFee and Das 1981; Wynn et al. 1975; Wahlstrom and Gustafsson 2014; McGary 2009; Birsan 2011; Song et al. 2014; Liu and Wang 2010). We can locate magnetic object like UXO using either a single magnetic sensor or sensor array with a designed scan routine (scan several lines) (Abdelrahman and Essa 2015; McFee and Das 1981). We can also use the magnetic contour map to locate the position of static magnetic object.

We can estimate a magnetic source with six parameters, three describing the position and three describing the magnetic moments of a target. The vector magnetic sensor can measure three components of the magnetic field. Thus, we can build three functions using one vector sensor. In order to calculate six parameters, at least two vector sensors are required. Thus, an array with magnetic vector sensors is widely used for locating or tracking position of moving object (Wahlstrom and Gustafsson 2014; Liu and Wang 2010; Song et al. 2014; Marschner and Fischer 2007; Nara et al. 2006). Nara et al. (2006) designed a magnetic sensor array for detecting the object position using the magnetic field and spatial gradients. Marschner and Fischer (2007) measured the magnetic object using a hall sensor array. Song et al. (2014) proposed the tracking method based on tri-axial transmitting coils and uniaxial sensing of the generated electromagnetic field. When the vector magnetic sensors are assembled into an array, they have a common alignment with the coordinate axes. If not, there will be large measurement error when alignment error of vector magnetic sensors exists (Sui et al. 2012). More important, it is difficult for us to deal with the alignment of vector sensor in the array.

However, scalar magnetic sensor such as optical pumped magnetometer is relatively insensitive to its orientation. In the theory of optical pumped magnetometer, the angle between the direction of the optical axis and the direction of the ambient field is called tumble angle *q*. The sensor can’t be operating only when the optical axis is parallel with the ambient field (*q* = 0° or *q* = 180°) or perpendicular to the ambient field (*q* = 90°). The optical pumped magnetometer will perform satisfactorily when the angle *q* within a range, like 10° < *q* < 85° or 95° < *q* < 170° (CS-L, Scintrex), 6° < *q* < 84° or 96° < *q* < 174° (G882, Geometrics). Thus, the measurement value of it is almost not influenced by its orientation in measurement coordinate axes. Therefore, it has a great advantage to assemble an array with scalar magnetic sensors. In this paper, we propose a method based on a scalar magnetometer array to track the magnetic target. The position of the target can be obtained by the proposed method in real time. In order to compute in real time, we use the particle swarm optimization (PSO) algorithm. In addition, we can estimate the magnetic moment of the target after computing its position.

## Localization theory

In the process of localization or track, the basic assumption is that the target can be modeled as a magnetic dipole. When the distance between the target and a sensors is three times longer than the size of itself, we can consider the target as the magnetic dipole (Wiegert and Gerovska 2000). The external magnetic field induced by the dipole can be described as:

1 $${\vec{\text{B}}}_{A} = \left| {\begin{array}{*{20}c} {B_{Ax} } \\ {B_{Ay} } \\ {B_{Az} } \\ \end{array} } \right| = \frac{{\mu_{0} }}{{4\pi r^{5} }}\left[ {\begin{array}{*{20}c} {3x^{2} - r^{2} } &\quad {3xy} &\quad {3xz} \\ {3xy} &\quad {3y^{2} - r^{2} } &\quad {3yz} \\ {3xz} &\quad {3yz} &\quad {3z^{2} - r^{2} } \\ \end{array} } \right]\left[ {\begin{array}{*{20}c} {M_{x} } \\ {M_{y} } \\ {M_{z} } \\ \end{array} } \right]$$

where *r* is the distance from the dipole with coordinates (0, 0, 0) to a sensor with coordinates (*x*, *y*, *z*). *M*_*x*_, *M*_*y*_, *M*_*z*_ denote the components of the magnetic moment $\vec{M}$ of the dipole. *μ*_0_ is the permeability.

In practice, the magnetic field measured by the sensor includes: the earth magnetic field $\vec{B}_{E}$ and the external magnetic field $\vec{B}_{A}$. However, $\vec{B}_{A}$ may vary from approximately 0.01–100 nT and is much smaller than $\vec{B}_{E}$. Therefore, it is valid that $\vec{B}_{A} \ll \vec{B}_{E} .$ And the magnetic anomaly Δ*B* generated by the magnetic target can be regarded as the projection of $\vec{B}_{A}$ on $\vec{B}_{E}$ and defined as (Stavrev and Gerovska 2000; Blakely 1996):

2 $$\begin{aligned} \Delta B & = \left| {{\vec{\text{B}}}_{\text{E}} + {\vec{\text{B}}}_{\text{A}} } \right| - \left| {{\vec{\text{B}}}_{\text{E}} } \right| = B_{m} - B_{E} \approx {\vec{\text{u}}} \cdot {\vec{\text{B}}}_{\text{A}} \\ & = B_{Ax} \cos (I_{0} )\cos (D_{0} ) + B_{Ay} \cos (I_{0} )\sin (D_{0} ) + B_{Az} \sin (I_{0} ) \\ \end{aligned}$$

where *B*_*m*_ is the magnetic sensor output value. ${\vec{\text{u}}}$ denotes the direction of vector $\vec{B}_{E} .$*I*_0_ and *D*_0_ denote the inclination and declination of the normal geomagnetic field, respectively.

Δ*B* can be expressed in matrix form as:

3 $$\Delta B = \frac{{\mu_{0} }}{{4\pi r^{5} }}{\mathbf{GKM}}$$

where $${\mathbf{G}} = [\begin{array}{*{20}l} {\cos (I_{0} )\cos (D_{0} )} \hfill & {\cos (I_{0} )\sin (D_{0} )} \hfill & {\sin (I_{0} )} \hfill \\ \end{array} ]$$, $${\mathbf{K}} = \left[ {\begin{array}{*{20}c} {3x^{2} - r^{2} } & {3xy} & {3xz} \\ {3xy} & {3y^{2} - r^{2} } & {3yz} \\ {3xz} & {3yz} & {3z^{2} - r^{2} } \\ \end{array} } \right]$$, $${\mathbf{M}} = \left[ {\begin{array}{*{20}c} {M_{x} } \\ {M_{y} } \\ {M_{z} } \\ \end{array} } \right].$$

We can separate the position and magnetic moment of the target by matrix transformation through Eq. (3). Then the expression is given by:

4 $${\mathbf{M}}^{\text{T}} \left( {{\mathbf{MM}}^{\text{T}} } \right)^{ - 1} {\mathbf{G}}^{\text{T}} = \frac{{\mu_{0} }}{{4\pi r^{5} \Delta B}}{\mathbf{GKG}}^{\text{T}}$$

We can see from Eq. (4) that ${\mathbf{M}}^{\text{T}} ({\mathbf{MM}}^{\text{T}} )^{ - 1} {\mathbf{G}}^{\text{T}}$ is a function of *M*_*x*_, *M*_*y*_, *M*_*z*_, *I*_0_, *D*_0_ and $\frac{{\mu_{0} }}{{4\pi r^{5} \Delta B}}{\mathbf{GKG}}^{\text{T}}$ is a function of *x*, *y*, *z*, *I*_0_, *D*_0_, Δ*B*. The magnetic moment information (*M*_*x*_, *M*_*y*_, *M*_*z*_) locates at the left side of Eq. (4) and the position information (*x*, *y*, *z*) locates at the right side of Eq. (4).

**Fig. 1:**
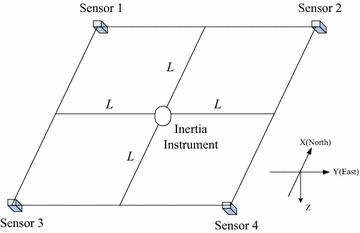
Schematic diagram of the scalar magnetometer array

Based on the analysis, we design an array with four scalar magnetometers and an inertia instrument, shown in Fig. 1. The inertia instrument is fixed at the array center. L is the distance between the array center to the boundary.

We can see from Eq. (4) that ${\mathbf{M}}^{\text{T}} ({\mathbf{MM}}^{\text{T}} )^{ - 1} {\mathbf{G}}^{\text{T}}$ is same for four scalar magnetometers at each measurement. Thus we can obtain the equation as:

5 $$\frac{{\mu_{0} }}{{4\pi r_{1}^{5} \Delta B_{1} }}{\mathbf{GK}}_{1} {\mathbf{G}}^{\text{T}} = \cdots = \frac{{\mu_{0} }}{{4\pi r_{4}^{5} \Delta B_{4} }}{\mathbf{GK}}_{4} {\mathbf{G}}^{\text{T}}$$

where $${\mathbf{K}}_{1} = \left[ {\begin{array}{*{20}c} {3(x + L)^{2} - r_{1}^{2} } &\quad {3(x + L)(y - L)} &\quad {3(x + L)z} \\ {3(x + L)(y - L)} &\quad {3(y - L)^{2} - r_{1}^{2} } &\quad {3(y - L)z} \\ {3(x + L)z} &\quad {3(y - L)z} &\quad {3z^{2} - r_{1}^{2} } \\ \end{array} } \right],$$$${\mathbf{K}}_{2} = \left[ {\begin{array}{*{20}c} {3(x + L)^{2} - r_{2}^{2} } &\quad {3(x + L)(y + L)} &\quad {3(x + L)z} \\ {3(x + L)(y + L)} &\quad {3(y + L)^{2} - r_{2}^{2} } &\quad {3(y + L)z} \\ {3(x + L)z} &\quad {3(y + L)z} &\quad {3z^{2} - r_{2}^{2} } \\ \end{array} } \right],$$$${\mathbf{K}}_{3} = \left[ {\begin{array}{*{20}c} {3(x - L)^{2} - r_{3}^{2} } &\quad {3(x - L)(y - L)} &\quad {3(x - L)z} \\ {3(x - L)(y - L)} &\quad {3(y - L)^{2} - r_{3}^{2} } &\quad {3(y - L)z} \\ {3(x - L)z} &\quad {3(y - L)z} &\quad {3z^{2} - r_{3}^{2} } \\ \end{array} } \right],$$$${\mathbf{K}}_{4} = \left[ {\begin{array}{*{20}c} {3(x - L)^{2} - r_{4}^{2} } &\quad {3(x - L)(y + L)} &\quad {3(x - L)z} \\ {3(x - L)(y + L)} &\quad {3(y + L)^{2} - r_{4}^{2} } &\quad {3(y + L)z} \\ {3(x - L)z} &\quad {3(y + L)z} &\quad {3z^{2} - r_{4}^{2} } \\ \end{array} } \right].$$

If the parameters—*I*_0_, *D*_0_, Δ*B*_*i*_—are known, the position of the target can be obtained by minimizing:

6 $$F(x,y,z) = \sum\limits_{i,j}^{4} {\left( {\frac{{\mu_{0} }}{{4\pi r_{i}^{5} \Delta B_{i} }}{\mathbf{GK}}_{i} {\mathbf{G}}^{\text{T}} - \frac{{\mu_{0} }}{{4\pi r_{j}^{5} \Delta B_{j} }}{\mathbf{GK}}_{j} {\mathbf{G}}^{\text{T}} } \right)}^{2}$$

In order to obtain the position of the target, a few assumptions of minor restrictiveness should be made. We assume that the geomagnetic field is constant or smoothly changes and its gradient remains very uniform in the measurement region (McFee and Das 1981). Δ*B*_*i*_ can be easily calculated by Δ*B*_*i*_ ≈ *B*_*mi*_ − *B*_*E*_, when *B*_*E*_ is known. *B*_*E*_ can be measured through the method in paper (McFee and Das 1981). Therefore, we can calculate the position of the target through Eq. (6) using some algorithms.

## Particle swarm optimization

In order to calculate the position of the target in real time, we use the PSO algorithm to obtain the solution, which can be rapidly converged and has few adjustable parameters (Eberhart and Kennedy 1995; Yang et al. 2010). In order to better understand the PSO algorithm, the detailed descriptions of some key terms in PSO are given as follows.

1. *Particle* A particle is an individual in the swarm. The position of each particle is adjusted by the velocity of them.
2. *Position* The position of each particle represents the candidate solution for the problem.
3. *Velocity* The direction and magnitude of the velocity determine the position of the particle in next iterative process. And the velocity of particle is changed according to the relative position of the personal best (pbest) and the global best (gbest).
4. *pbest* The pbest is a position with the best fitness value discovered by a particle in the solution space.
5. *gbest* The gbest is a position with the best fitness value discovered by the entire swarm in the solution space.
6. *Fitness* The fitness is a value of the fitness function with one solution. And it can represent the quality of the solution.
7. *Solution space* The solution space is a reasonable range in which the particles search for the optimal solution.
8. *Fitness function* The fitness function is a mathematical expression of the problem and is used to evaluate the position of each particle.

The PSO algorithm works on social behavior of particles in the swarm. Particles fly around in solution space. And in each iterative process, the position of each particle in the solution space is adjusted by dynamically altering the velocity of each particle, according to its own experience and the experience of other particles (Robinson and Rahmat-Samii 2004; Ratnaweera et al. 2004). Therefore, in the PSO algorithm, the *i*th particle is described by position vector $x_{i} = (x_{i1} ,x_{i2} , \ldots ,x_{id} )$ and velocity vector $v_{i} = (v_{i1} ,v_{i2} , \ldots ,v_{id} )$ and *d* is the dimension of the solution space. According to the fitness function defined by user, the previous best position of the *i*th particle $P_{i} = (p_{i1} ,p_{i2} , \ldots ,p_{id} )$ is the best fitness value obtained by that particle. And the previous best position of the group $P_{g} = (p_{g1} ,p_{g2} , \ldots ,p_{gd} )$ is the best fitness value obtained by swarm. In each iterative process, the velocity and the position of a particle are updated according to the following equations:

7 $$v_{ij} (t + 1) = wv_{ij} + c_{1} rand_{1} \left( {p_{ij} (t) - x_{ij} (t)} \right) + c_{2} rand_{2} \left( {p_{gj} (t) - x_{ij} (t)} \right)$$

8 $$x_{ij} (t + 1) = x_{ij} (t) + v_{ij} (t + 1)$$

where *c*_1_ and *c*_2_ are acceleration factors, *rand*_1_ and *rand*_2_ are uniform random variables in the interval [0, 1]. *w* is the inertia weight.

Much work has been done to understand and develop the ideal parameters for PSO implementation. Eberhart and Shi (2001), Shi and Eberhart (1999) suggested varying the value of *w* from 0.9 at the beginning of search to 0.4 at the end of search and suggested that the best value of *c*_1_ and *c*_2_ is 1.49 in most problems. In addition, population size *N* is also an important parameter and should be selected carefully. Large size increases the execution time and reduces the efficiency of the algorithm. While, small size leads to low accuracy of the optimal solution. Parametric studies on the size (Shi and Eberhart 1998; Ratnaweera et al. 2004) have found that the size should be selected in the range from 10 to 60. And the best value of the population size should be determined according to the problem.

## Experimental section

We conducted two simulation experiments in this section. In the first, we determined the best value of the population size of the PSO algorithm. And in the second, we tested the performance of the proposed method using the PSO algorithm. In the two experiments, the magnetic moment of the target was set [920, −102, 1100] A m^2^. The length L of the array was 3 m. The solution space of the problem was set $\{ [ - 100,\,100]{\kern 1pt} {\kern 1pt} ;\,[ - 100,\,100]{\kern 1pt} {\kern 1pt} ;\,[10,\,50]\} {\text{m}}$. Scalar magnetometers is with high sensitivity, and intrinsic noise was about 0.6 ${\text{pT/}}\sqrt {\text{Hz}}$ at 1 Hz. Geomagnetic field measurement error was set as: the average is zero and the standard deviation is 0.1 nT.

## Results and discussion

### Population size of the PSO algorithm

In the first experiment, the magnetic target was at a fixed position and we changed the population size of the PSO algorithm to calculate the position of the target. The algorithm ran 100 times at each population size, and the results were shown in Table 1.

**Table 1:** Effect of population size on the algorithm

Population size	RMSE			Execution time (s)
	X position	Y position	Z position	
10	1.14053	1.60896	0.72492	0.19503
20	0.92803	1.30975	0.50881	0.37247
30	0.53957	0.67759	0.34209	0.56457
40	0.54759	0.64686	0.34652	0.75011
50	0.52929	0.6308	0.33388	0.95753
60	0.53449	0.60565	0.32572	1.12175

In Table 1, we can see that the calculation accurate and execution time of the algorithm is related to the population size. The calculation accurate increases along with the size. And the execution time also increases along with the size. However, we find that the accurate is almost not improved when the size reaches a certain level. Thus, the population size of the PSO algorithm is 30.

### Track results

**Fig. 2:**
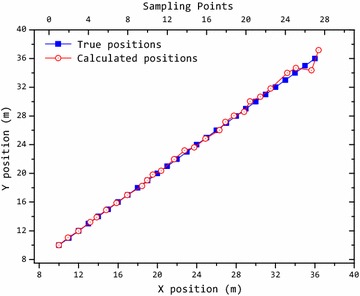
Localization result of magnetic target

**Fig. 3:**
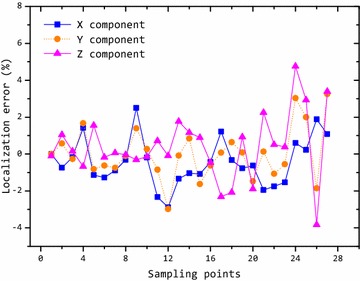
Relative error of localization of magnetic target

In the second experiment, the target moved along a plan trajectory. And we used the PSO algorithm to locate the target. The localization result is shown in Fig. 2. It shows that the tracked trajectory obtained by this method is close to the plan trajectory. And the positions of the target are calculated accurately. Figure 3 shows the relative error of the position component at each sampling point. It can be seen that the localization error increases as the distance increases, because the useful magnetic anomaly information attenuates with three cubed function. The relative error of localization is <5 %. Statistically, the localization error will be larger when the distance becomes larger.

The magnetic moment of the target can be estimated when the target’s position had been calculated, as:

9 $$\left[ {\begin{array}{*{20}c} {M_{x} } \\ {M_{y} } \\ {M_{z} } \\ \end{array} } \right] = \left[ {\begin{array}{*{20}c} {\frac{{\mu_{0} }}{{4\pi r_{1}^{5} }}{\mathbf{GK}}_{1} } \\ {\frac{{\mu_{0} }}{{4\pi r_{2}^{5} }}{\mathbf{GK}}_{2} } \\ {\frac{{\mu_{0} }}{{4\pi r_{3}^{5} }}{\mathbf{GK}}_{3} } \\ \end{array} } \right]^{ - 1} \left[ {\begin{array}{*{20}c} {\Delta B_{1} } \\ {\Delta B_{2} } \\ {\Delta B_{3} } \\ \end{array} } \right]$$

where *M*_*x*_, *M*_*y*_, *M*_*z*_ denote the component of the magnetic moment $\vec{M}$ of the target.

**Fig. 4:**
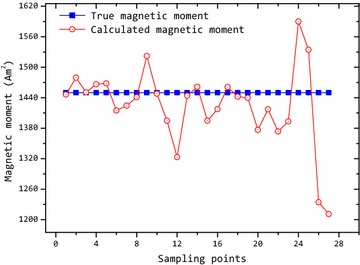
Magnetic moment of the target

Scalar value of magnetic moment can be obtained by Eq. (9), which is related to the target size. We can also estimate the orientation of the magnetic target by magnetic moment (*M*_*x*_, *M*_*y*_, *M*_*z*_). Figure 4 shows the magnetic moment of the target calculated by Eq. (9). Comparing the theoretical value and the calculated value of moment, there is a difference between the values. It is mainly because that the calculated position of the target is not accuracy. Therefore, the accurate of calculated moment depends on the accurate of calculated position. From (9), we can know that the error of calculated moment comes from the error of the target’s position.

**Fig. 5:**
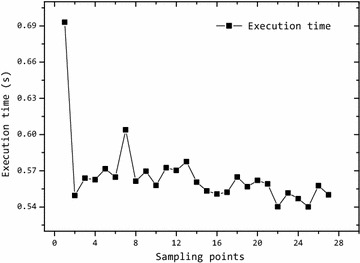
Execution time of the PSO algorithm

In addition, we also investigate the execution time of the PSO algorithm. Figure 5 shows the execution time of the PSO algorithm. The maximum time is about 0.69 s and the average time is about 0.56 s. The execution time is possible for the method to track the target in real-time.

### Discussion

Equation (4) is obtained in the case that the array is in the static state. Consider using a magnetometer array on a moving vehicle and trying to locate a magnetic target. Rotational vibrations due to the vehicle’s motion will generate changes of the array attitude. Therefore, the position (*x*, *y*, *z*) in Eq. (4) should be corrected by the attitude angles. In our magnetometer array, the attitude angles (*α*, *β*, *γ*) of the array can be measured by the inertia instrument. And the corrected position of the target is expressed as:

10 $$\left( {x^{\prime } ,y^{\prime } ,z^{\prime } } \right) = (x,y,z){\mathbf{R}}_{\alpha } {\mathbf{R}}_{\beta } {\mathbf{R}}_{\gamma }$$

where $${\mathbf{R}}_{\alpha } = \left[ {\begin{array}{*{20}c} 1 &\quad 0 &\quad 0 \\ 0 &\quad {\cos \,\alpha } &\quad {\sin \,\alpha } \\ 0 &\quad { - \sin \,\alpha } & {\cos \,\alpha } \\ \end{array} } \right],$$$${\mathbf{R}}_{\beta } = \left[ {\begin{array}{*{20}c} {\cos \,\beta } &\quad 0 &\quad { - \sin \,\beta } \\ 0 &\quad 1 &\quad 0 \\ {\sin \,\beta } &\quad 0 &\quad {\cos \,\beta } \\ \end{array} } \right],$$$${\mathbf{R}}_{\gamma } = \left[ {\begin{array}{*{20}c} {\cos \,\gamma } &\quad {\sin \,\gamma } &\quad 0 \\ { - \sin \,\gamma } &\quad {\cos \,\gamma } &\quad 0 \\ 0 &\quad 0 &\quad 1 \\ \end{array} } \right].$$

**Fig. 6:**
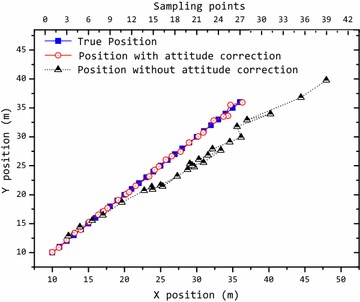
Localization result of the magnetic target with the rotation of the array

In this simulation, the array attitude angles were changed and measured by the inertia instrument: (*α* = 5°, *β* = 10°, *γ* = 5°). The target moved along a plan trajectory. And we used the PSO algorithm to locate the target. The localization result is shown in Fig. 6. When the array attitude is changed, the calculated position with attitude correction is close to the true position. However, the calculated position without attitude correction has a large difference from the true position. Therefore, we must correct the position (*x*, *y*, *z*) in Eq. (4) by using the array attitude angles if its attitude changes.

In the PSO algorithm, the fitness function is a link between the particles and the physical world. And the well fitness function should have a good performance both in the quality of the solution and in the convergence speed. Thus, well fitness function must fulfill two criteria. First, the fitness function should be sensitive enough to the global optimal solution. In other words, it should be a steep function of the variables when the variables are close to the global optimal solution, otherwise it should be a smooth function of the variables. In our fitness function [Eq. (6)], it is a steep function when the variables are close to the global optimal solution. However, there are some local optima in the solution space. Sometimes, the PSO algorithm can’t jump out from the local optima when trapping in it. This is the reason why the quality of the solution calculated by the PSO algorithm is not too high. Second, the fitness function should be simple enough to reduce calculating time. In our fitness function [Eq. (6)], it is relatively complex and consumes more computing time. Therefore, we will improve the PSO algorithm ability of finding the global optimal solution and construct a better fitness function in the future.

## Conclusions

In this paper, we propose an efficient method for tracking the magnetic target in real-time, which consists of scalar magnetometers array and a PSO algorithm. The scalar magnetometers are used to measure the scalar value of the magnetic anomaly included by the magnetic target. We separate the position information and magnetic moment information by matrix transformation and build the function *F* of x, y and z. Then, we use the PSO algorithm to obtain the solution of the function *F*. The simulation result shows that the position of the magnetic target can be calculated accurately. Then the magnetic moment of the target can be estimated when the target’s position had been calculated. The PSO algorithm can reduce the execution time. Therefore, this method can be used for real time localization of the magnetic target. In addition, because of the array formed by scalar magnetometers which are insensitive to its orientation, it is easy for this array to be mounted on the platform.
